# Caspase-1 inhibition improves cognition without significantly altering amyloid and inflammation in aged Alzheimer disease mice

**DOI:** 10.1038/s41419-022-05290-x

**Published:** 2022-10-11

**Authors:** Joseph Flores, Marie-Lyne Fillion, Andréa C. LeBlanc

**Affiliations:** 1 Lady Davis Institute for Medical Research at Jewish General Hospital, Montréal, QC Canada; 2 Department of Neurology and Neurosurgery, McGill University, Montréal, QC Canada

**Keywords:** Cell death in the nervous system, Alzheimer's disease, Hippocampus, Alzheimer's disease

## Abstract

Human genetic and animal model studies indicate that brain microglial inflammation is a primary driver of cognitive impairment in Alzheimer Disease (AD). Inflammasome-activated Caspase-1 (Casp1) is associated with both AD microglial inflammation and neuronal degeneration. In mice, *Casp1* genetic ablation or VX-765 small molecule inhibition of Casp1 given at onset of cognitive deficits strongly supports the association between microglial inflammation and cognitive impairment. Here, VX-765 significantly improved episodic and spatial memory impairment eight months after the onset of cognitive impairment in aged AD mice with significant amyloid beta peptide (Aβ) accumulation and microglial inflammation. Unexpectedly, while cognitive improvement was associated with dendritic spine density and hippocampal synaptophysin level recovery, VX-765 only slightly decreased Aβ deposition and did not alter biochemically-measured Aβ levels. Furthermore, increased hippocampal Iba1^+^-microglia, GFAP^+^-astrocytes, IL-1β, and TNF-α levels were unaltered by VX-765. These results support the hypothesis that neuronal degeneration, not Aβ or microglial inflammation, drives cognitive impairment in AD.

## Introduction

Aging and Alzheimer disease (AD) cognitive impairment is the result of hippocampal neuronal dysfunction. Therefore, targeting the molecular pathways underlying hippocampal neurodegeneration, defined here as the loss of neuron structure or function before cell death, could represent an efficient treatment strategy against memory loss associated with aging and AD. Furthermore, this approach would bypass the initial trigger(s) of cognitive impairment.

Research with human primary neuron cultures to identify a molecular pathway of neurodegeneration associated with cognitive impairment has revealed that the sequential activation of inflammasome Nucleotide-binding oligomerization domain, Leucine rich Repeat and Pyrin domain containing 1 (Nlrp1), Caspase-1 (Casp1), and Caspase-6 (Casp6) by serum deprivation or over-expression of wild-type (WT) and familial AD-associated mutant amyloid precursor protein (APP) is associated with amyloid-beta (Aβ)-independent loss of neuron structure and function and Aβ-dependent neuronal cell death [1–4]. Neurodegeneration via this pathway may be the result of the propensity for active Casp6 to cleave several cytoskeletal and synaptic neuronal proteins, and cleavage of valosin-containing protein 97, thereby promoting cellular accumulation of misfolded proteins [5, 6].

In vivo activation of the Nlrp1-Casp1-Casp6 pathway was confirmed in WT, *Nlrp1*^*-/-*^ and *Casp1*^*-/-*^ mouse brains with lipopolysaccharide-induced inflammation [1]. Expression of a self-activated form of Casp6 in mouse hippocampal CA1 neurons induces age-dependent cognitive impairment [7]. Furthermore, active Casp6 impairs synaptic transmission and induces neurodegeneration in acute brain slice hippocampal CA1, but not striatal medium spiny, neurons [3]. Casp6-mediated loss of synaptic function and cognitive impairment is reversable with general caspase inhibitors [4, 8–10], indicating that Casp6 activity does not cause neuronal cell death, but loss of neuronal structure and function

The implication of the Nlrp1-Casp1-Casp6 neurodegenerative pathway in cognitive impairment was demonstrated in the APP^Swedish/Indiana^ (APP^Sw/Ind^) transgenic J20 AD mouse model. Genetically ablating the *Nlrp1*^*-/-*^, *Casp1*^*-/-*^, or *Casp6*^*-/-*^ genes in J20 prevents the onset of both episodic and spatial memory deficits [11, 12]. Knockout [13] or siRNA knockdown [14] of *Casp1* improves cognition in APP/PS1 mice. Furthermore, pre-symptomatic treatment of J20 with the non-toxic, blood-brain barrier permeable, small molecule Casp1 inhibitor, VX-765, delays episodic and spatial memory deficits and hippocampal microglial inflammation for at least 5 months without altering Aβ levels [15–17]. VX-765 given at onset of J20 cognitive impairment normalizes cognition, synaptic markers, Iba1^+^-microglia and decreases Aβ levels [12].

Nlrp1, Casp1, and Casp6 are increased in human AD brains and associated with cognition. A 20-fold increase of Nlrp1-positive neurons, often containing active Casp6, was observed in both familial and sporadic AD brains [1]. Active Casp1 is increased in AD hippocampus and cortex [13]. Active Casp6 and Tau cleaved by Casp6 (Tau∆Casp6) are abundantly present in neuritic plaques, neurofibrillary tangles, and neuropil threads of sporadic and familial AD brains [18, 19]. Cerebrospinal fluid Tau∆Casp6 correlate with AD severity and cognitive status [20]. Casp6 activity in the anterior olfactory nuclei, from which some neurons project to the entorhinal cortex, also correlate with cognitive status [21]. In aged non-cognitively impaired individuals, increased Casp6 activation in the entorhinal cortex and hippocampal CA1 is associated with lower episodic and semantic memory performance, brain regions and memory types initially affected in AD [22, 23].

The importance of microglia in AD pathophysiology is supported by genetic association of several immune proteins with AD [24]. Furthermore, Aβ-mediated microglial Nlrp3 inflammasome-activated Casp1 is involved in disease burden of APP/PS1 mice [13, 25–28]. VX-765 treatment in J20 normalizes Iba1^+^-microglial levels [12, 17], and J20 *Casp1* knockout normalizes Iba1^+^-microglia [12] and pro-inflammatory cytokines TNF-α and CXCL1 levels [11]. Furthermore, novel object recognition (NOR) performance correlates significantly with Iba1^+^-microglia levels [17]. However, our findings do not entirely support microglia as a pivotal player in Nlrp1-Casp1-Casp6-mediated cognitive impairment. Some data support neurodegeneration, not microglial inflammation, as the cause of cognitive impairment. For example, TNF-α and CXCL1 are expressed in both neurons and microglia. Furthermore, the main Casp1 substrate, interleukin-1-beta (IL-1β), which is highly produced in activated microglia, was physiologically low, if detected, and minimally affected by VX-765 in J20 hippocampus and cortex, despite VX-765 normalizing cognition [11, 12, 17]. These results suggest that Casp1 activation in neurons produce considerably lower levels of IL-1β, than in microglia [1]. Since J20 mice, in these studies, reached 8 months of age at most, it is possible that low Aβ levels in 4- to 8-month-old J20 mice were not sufficient to fully activate the microglial Nlrp3-Casp1 pathway observed in 16-month-old APP/PS1 mice [13]. In the absence of Aβ-mediated microglial activation, increased Iba1^+^-microglial levels could simply be initiated by degenerating neurons [11].

To assess the effect of VX-765 in a brain with significant Aβ accumulation, 12-month-old J20 mice were treated with a VX-765 regimen identical to that previously used on 5-month-old mice [12]. Our results demonstrate that while cognition, dendritic spine density and synaptophysin levels are restored in VX-765-treated older J20 mice, VX-765 did not reduce increased Iba1^+^-microglia, Il-1β, TNF-α, GFAP^+^-astrocytes, or Aβ. These results indicate that episodic and spatial memory deficits in the J20 mice are unlikely caused by Aβ or Aβ-induced microglial activation.

## Results

**Fig. 1:**
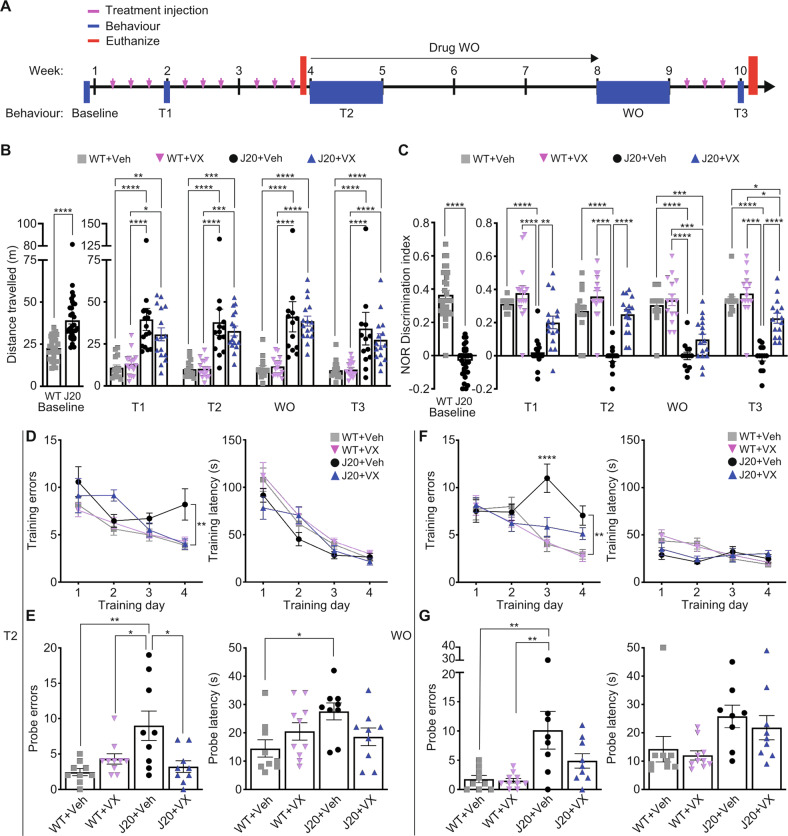
VX-765 reverses cognitive deficits in 15-month-old J20 mice. **A** Experimental treatment and behavioural paradigm illustrating treatment injections (pink), behavioural time points (blue), and euthanasia for post-mortem analyses (red). **B** Distance travelled in open field task at baseline [*n* = 31 WT, *n* = 32 J20; *t* = 6.44, *p* < 0.0001, unpaired *t*-test] and in vehicle (Veh) or VX-765 (VX)-treated mice at T1 (3 injections), T2 (9 total injections), WO (1 month washout), and T3 (12 total injections) [*n* = 15 WT + Veh, 16 WT + VX, 16 J20 + Veh, and 16 J20 + VX; F _time-point_ = 6.83, *p* = 0.0002; F _treatment_ = 13.28, *s* < 0.0001, two-way ANOVA, Bonferroni’s post-hoc]. **C** Novel object recognition (NOR) discrimination index at baseline [*t* = 13.22, *p* < 0.0001, unpaired t-test] and at T1, T2, WO, and T3 [F _treatment_ = 95.18, *p* < 0.0001, two-way ANOVA, Bonferroni’s post-hoc]. Number of mice per group are indicated in (**B**). **D**–**G** Barnes maze behaviour of *n* = 9 WT + Veh, 10 WT + VX, 9 J20 + Veh, and 9 J20 + VX at T2 (**D,E**) and *n* = 9 WT + Veh, 10 WT + VX, 8 J20 + Veh, and 9 J20 + VX after 1-month WO (**F**, **G**). **D**, **F** Barnes maze learning acquisition showing primary errors (left) and primary latency (right) to reach the target during the 4-day training period at (**D**) T2 [Training errors: *F*_training-day_ = 15.41, *p* < 0.0001; *F*_treatment_ = 4.67, *p* = 0.007, two-way repeated-measures ANOVA, Bonferroni’s post-hoc compared to WT + Veh] and (**F**) WO [Training errors: *F*_training-day_ = 12.81, *p* < 0.0001; *F*_treatment_ = 5.63, *p* = 0.003; *F*_interaction_ = 4.25, *p* = 0.0001; *F*_subject_ = 1.73, *p* = 0.02, two-way repeated measures ANOVA, Bonferroni’s post-hoc compared to WT + Veh]. **E**, **G** Barnes maze probe showing primary errors (left) and primary latency (right) to blocked target at (**E**) T2 [errors: *F*_genotype_ = 5.391, *p* = 0.03; F_interaction_ = 10.47, *p* = 0.003; latency: *F*_interaction_ = 6.00, *p* = 0.02, two-way ANOVA, Bonferroni’s post-hoc] and (**G**) WO [errors: *F*_genotype_ = 13.43, *p* = 0.0009; latency: *F*_genotype_ = 8.28, *p* = 0.007, two-way ANOVA, Bonferroni’s post-hoc]. Bars represent mean ± SEM; symbols denote performances of individual mice. *p < 0.05, **p < 0.01, ***p < 0.001, ****p < 0.0001.

Twelve-month-old J20 and WT mice were behaviourally assessed multiple times using a protocol previously implemented in younger 5-month-old J20 mice [12]: at baseline comparing WT and J20 prior to treatment, after three injections/week (T1) of 50 mg • kg^−1^ VX-765 or vehicle, after an additional two weeks of three injections/week (T2), after four weeks of drug washout (WO) with no injections, and after a final week of three injections (T3) (Fig. 1A). A total of 12 treatment injections was given to each mouse spanning three months. Mice were euthanized at 14.5–15 months of age and examined with biochemical and immunohistological methods. A subset of animals, which did not undergo behavioural testing, were euthanized after nine injections at T2 for Golgi-Cox analysis.

### VX-765 reverses episodic and spatial memory deficits, but not hyperactivity, in aged J20 mice

Open field was implemented to determine locomotor behaviour and hyperactivity in J20 versus WT mice. Compared to WT, untreated 12 month-old J20 (baseline) had increased distance travelled, # of quadrant entries and % time moving (Fig. 1B, Fig. S1A,B). These results support previous observations of J20 hyperactivity [29]. J20 did not show any difference from WT in % time spent in the periphery (thigmotaxis) indicating normal anxiety levels (Fig. S1C). This profile was maintained in vehicle-treated J20 from 12 to 15 months of age during T1, T2, WO and T3. VX-765 did not affect J20 or WT locomotor behaviour, hyperactivity, or thigmotaxis, although VX-765 was previously observed to reverse locomotor activity in 5-month-old J20 mice [12].

NOR was used to determine whether VX-765 can reverse APP^Sw/Ind^-induced episodic memory deficits in 12- to 15-month-old J20. J20 showed a strong deficit in NOR discrimination index (DI) compared to WT at baseline. J20 NOR DI improved to normal WT levels after VX-765 treatment at T1 and T2 (Fig. 1C). NOR deficits reappeared in VX-765-treated J20 and were no different than vehicle-treated J20 mice after 4-week WO but were partially rescued after additional VX-765 treatment at T3. VX-765 treatment did not affect WT mice at any of the testing periods.

Mice underwent the Barnes maze test at T2 and after WO to assess spatial learning and memory. At T2, vehicle-treated J20 committed significantly more errors to find the target than WT by the end of the four-day training period (Fig. 1D). In VX-765-treated J20, this learning deficit reverted to normal WT levels. Latencies to reach the target were the same across all treatment groups (Fig. 1D). During the T2 probe, vehicle-treated J20 committed more errors and were slower to find the target than WT. Spatial memory was normalized with VX-765 (Fig. 1E). Vehicle-treated J20 did not show preference for the target area compared to WT, and this was normalized with VX-765 (Fig. S2A). VX-765 did not affect spatial learning or probe memory in WT mice.

After WO, vehicle-treated J20 continued making more errors finding the target compared to WT at both day three and four during the training period, but errors were reduced in VX-765-treated J20 (Fig. 1F). Latencies to reach the target were much shorter compared to T2 and were the same across the training period and between all treatment groups (Fig. 1F). During the WO probe, vehicle-treated J20 continued to make significantly more errors than WT (Fig. 1G), but errors were reduced in VX-765-treated J20. Probe latency did not differ across the treatment groups. Consistently, all groups, except vehicle-treated J20, showed a preference for the target area (Fig. S2B). VX-765 did not affect WT spatial learning and memory at WO. These results indicate that VX-765 confers long-term reversal of spatial memory deficits in 13-month-old (T2) and 14.5-month-old (WO) J20 mice.

Together, these results indicate that VX-765 normalized both episodic and spatial memory after over 10 months of cognitive impairment in aged J20 mice.

### Decreased dendritic spine density and synaptophysin levels were recovered in VX-765-treated aged J20 mice

**Fig. 2:**
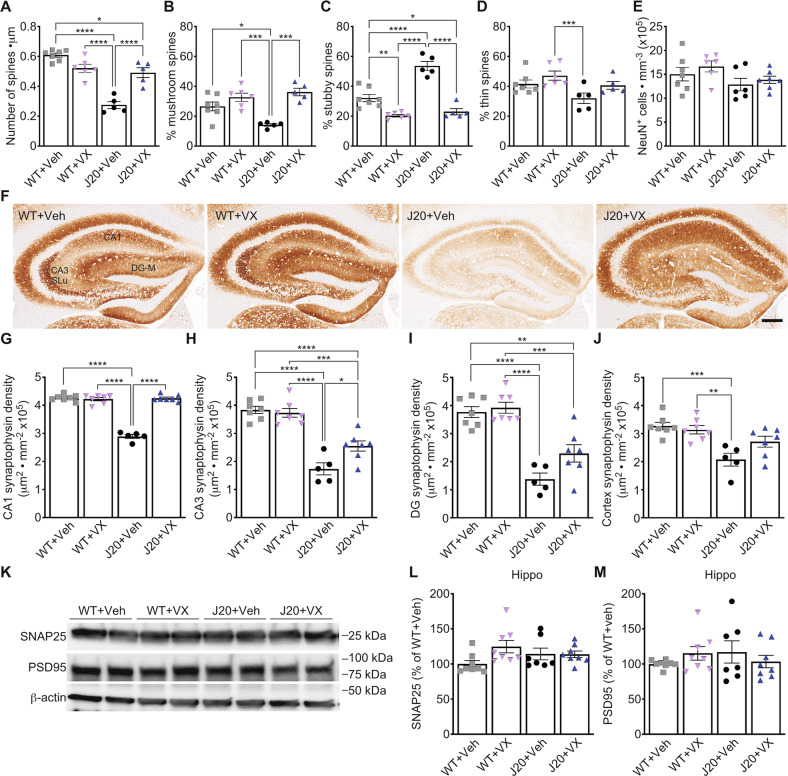
VX-765 normalizes dendritic spine density and synaptophysin levels in aged J20 hippocampal CA1. **A** Golgi-Cox dendritic spine density of *n* = 7 WT + Veh, 6 WT + VX, 5 J20 + Veh, and 5 J20 + VX mice [*F*_genotype_ = 56.49, *p* < 0.0001; *F*_treatment_ = 7.11, *p* = 0.02; *F*_interaction_ = 39.43, *p* < 0.0001] and percentage number of (**B**) mushroom [*F*_treatment_ = 28.85, *p* < 0.0001; *F*_interaction_ = 9.25, *p* < 0.007], **C** stubby [*F*_genotype_ = 29.22, *p* < 0.0001; *F*_treatment_ = 90.30, *p* < 0.0001; F _interaction_ = 17.73, *p* = 0.0004], and (**D**) thin [F _genotype_ = 7.30, *p* = 0.01; *F*_treatment_ = 5.65, *p* = 0.03] spine subtypes. **E** NeuN^+^ cell density in the hippocampal CA1 PCL [*F*_genotype_ = 4.40, *p* = 0.05]. **F** Representative synaptophysin immunostaining in the hippocampus. Separate density quantifications were made in the hippocampal CA1, stratum lucidum of the CA3 (CA3 SLu), and the molecular layer of the dentate gyrus (DG-M). Scale bar = 250 μm. **G**–**J** Synaptophysin staining density of *n* = 7 WT + Veh, 7 WT + VX, 5 J20 + Veh, and 7 J20 + VX mice in the (**G**) CA1 [*F*_genotype_ = 157.3, *p* < 0.0001; *F*_treatment_ = 150.7, *p* < 0.0001; *F*_interaction_ = 173.6, *p* < 0.0001], (**H**) CA3 [*F*_genotype_ = 92.38, *p* < 0.0001; *F*_treatment_ = 4.31, *p* = 0.05; *F*_interaction_ = 7.22, *p* = 0.01], (**I**) DG [F _genotype_ = 68.63, *p* < 0.0001; *F*_treatment_ = 4.85, *p* = 0.04], and (**J**) cortex [*F*_genotype_ = 21.67, *p* = 0.0001; F _interaction_ = 4.89, *p* = 0.04]. **K** Representative western blot images and quantification of hippocampal (**L**) SNAP25 and (**M**) PSD95 of *n* = 8 WT + Veh, 8 WT + VX, 7 J20 + Veh, and 8 J20 + VX mice. Two-way ANOVA, Bonferroni’s post-hoc for all panels. Bars represent mean ± SEM; symbols denote performances of individual mice. *p < 0.05, **p < 0.01, ***p < 0.001, ****p < 0.0001.

Dendritic spine density and morphology were measured with Golgi-Cox staining in the stratum radiatum (SR) of the hippocampal CA1 at T2 (Fig. S3A). Dendritic spine density (Fig. 2A) decreased approximately 55% in vehicle-treated J20 compared to WT. Spine morphology analyses revealed decreased mushroom (Fig. 2B) and increased stubby spine subtypes (Fig. 2C) in vehicle-treated J20 compared to WT. VX-765 considerably increased dendritic spine density, normalized mushroom spine levels and decreased stubby spine levels to near-WT levels in J20. The thin spine subtype in vehicle-treated J20 was decreased compared to VX-765-treated WT but no other differences were found (Fig. 2D). There were no differences in NeuN^+^ cell density in the CA1 pyramidal cell layer (PCL) between all groups (Fig. 2E), suggesting that changes in dendritic spine density was not due to neuronal loss.

Synaptophysin is important in NOR and spatial learning [30]. Synaptophysin staining density was measured in the hippocampal CA1, CA3, and dentate gyrus molecular layer (DG-M) (Fig. 2F) and in the cortical retrosplenial (RS) and adjacent motor and somatosensory areas (Fig. S3B) in 15-month-old J20. Synaptophysin density decreased in the hippocampal CA1, CA3 stratum lucidum (SLu), and DG-M in vehicle-treated J20 relative to WT (Fig. 2G–I). Synaptophysin density was normalized in the CA1 (Fig. 2G), partially restored in the CA3-SLu (Fig. 2H), and unaltered in the DG-M (Fig. 2I) of VX-765-treated J20. In the cortex, synaptophysin density decreased in J20 compared to WT and was partially restored with VX-765 (Fig. 2J). No changes in synaptophysin density were observed in the CA1, CA3, DG or cortex of VX-765-treated WT (Fig. 2G–J). The pre- and post-synaptic proteins, SNAP25 and PSD95, respectively, assessed with western blot (Fig. 2K), did not change between WT and J20 or after VX-765 (Fig. 2L, M). These results indicate that VX-765 normalization of hippocampal synaptophysin density may explain VX-765’s ability to normalize both NOR and spatial memory in aged J20 mice.

### VX-765 does not change increased Iba1^+^-microglia or GFAP^+^-astrocyte levels in 15-month-old J20 mice

**Fig. 3:**
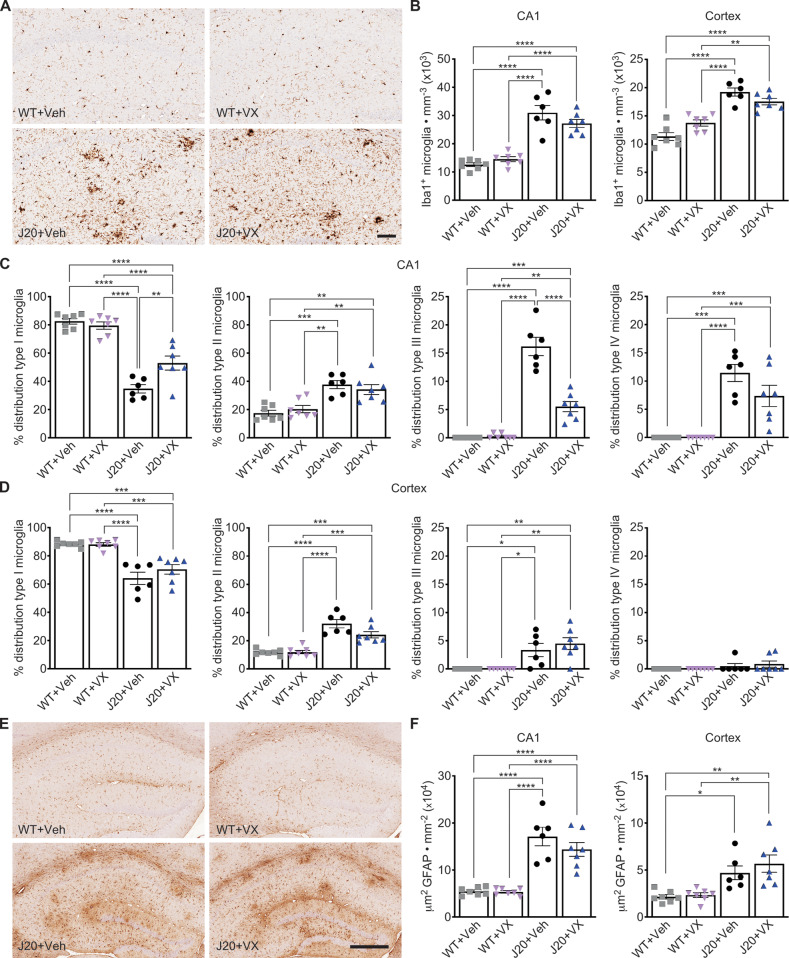
VX-765 does not change Iba1^+^-microglia or GFAP^+^-astrocyte levels in 15-month-old J20 mice. **A** Representative Iba1^+^ microglial immunostaining in the hippocampus. Scale bar = 100 μm. **B** Iba1^+^ immunostaining density in the hippocampal CA1 (left panel) of *n* = 7 WT + Veh, 7 WT + VX, 6 J20 + Veh, and 7 J20 + VX mice [*F*_genotype_ = 110.9, *p* < 0.0001] and cortex (right panel) [*F*_genotype_ = 85.91, *p* < 0.0001; *F*_interaction_ = 10.53, *p* = 0.004]. **C** Hippocampal morphological distribution of type I [*F*_genotype_ = 125.0, *p* < 0.0001; *F*_treatment_ = 5.19, *p* = 0.03; *F*_interaction_ = 10.19, *p* = 0.004], type II [*F*_genotype_ = 37.68, *p* < 0.0001], type III [*F*_genotype_ = 155.4, *p* < 0.0001; *F*_treatment_ = 36.58, *p* < 0.0001; *F*_interaction_ = 40.28, *p* < 0.0001], and type IV [F_genotype_ = 63.51, *p* < 0.0001] microglia. **D** Cortical morphological distribution of type I [*F*_genotype_ = 59.06, *p* < 0.0001], type II [*F*_genotype_ = 71.97, *p* < 0.0001; *F*_interaction_ = 4.39, *p* = 0.05], type III [*F*_genotype_ = 27.49, *p* < 0.0001], and type IV microglia. **E** Representative GFAP^+^ astrocyte immunostaining in the hippocampus. Scale bar = 400 μm. **F** GFAP^+^ immunostaining density in the hippocampal CA1 ^(^left panel) [*F*_genotype_ = 77.69, *p* < 0.0001] and cortex (right panel) [*F*_genotype_ = 23.76, *p* < 0.0001]. Number of mice as indicated in (**B**). Two-way ANOVA, Bonferroni’s post-hoc for all panels. Bars represent mean ± SEM; symbols denote performances of individual mice. *p < 0.05, **p < 0.01, ***p < 0.001, ****p < 0.0001.

Iba1^+^-microglia numbers were assessed in 15-month-old J20 mice hippocampal CA1 (Fig. 3A) and cortical RS and adjacent motor and somatosensory areas (Fig. S4A) to determine whether VX-765-dependent cognitive improvements were associated with changes in the inflammatory milieu. Compared to WT, Iba1^+^-microglia increased 2- and 1.5-fold in vehicle-treated J20 hippocampal CA1 and cortex (Fig. 3B), respectively, as previously observed in younger 8-month-old J20 [12]. In contrast to younger J20, increased Iba1^+^-microglia levels were maintained after VX-765 treatment in aged J20 (Fig. 3B). These results indicate that VX-765-mediated normalization of cognition in aged J20 is independent from Iba1^+^-microglia levels.

Microglia were assessed morphologically and separated into four different profiles based on ramified resting (type I), activated (type II), reactive (type III), and phagocytic (type IV) morphologies (Fig. S4B) [31]. In the CA1, vehicle-treated J20 had lower type I and higher type II, III, and IV microglial subtypes compared to WT (Fig. 3C). VX-765 increased type I and decreased type III microglia in J20, but WT levels were not reached. Type II and IV microglia were unaffected by VX-765. In the cortex, vehicle-treated J20 showed decreased type I, increased type II and III, and low unaltered type IV levels, compared to WT (Fig. 3D). VX-765 did not alter type I, II, III or IV microglia in WT or J20.

GFAP^+^-astrocyte staining density was measured in the hippocampal CA1 (Fig. 3E) and cortical RS and adjacent areas (Fig. S4C). Compared to WT, vehicle-treated J20 showed increased GFAP^+^ staining in the CA1 (Fig. 3F) and cortex (Fig. 3F), which was not affected with VX-765 in either WT or J20. These results indicate that improved cognition in J20 mice is not due to changes in astrogliosis during inflammation.

### VX-765 normalizes decreased IL-10, but not increased Il-1β and TNF-α, in 15-month-old J20 hippocampus

**Fig. 4:**
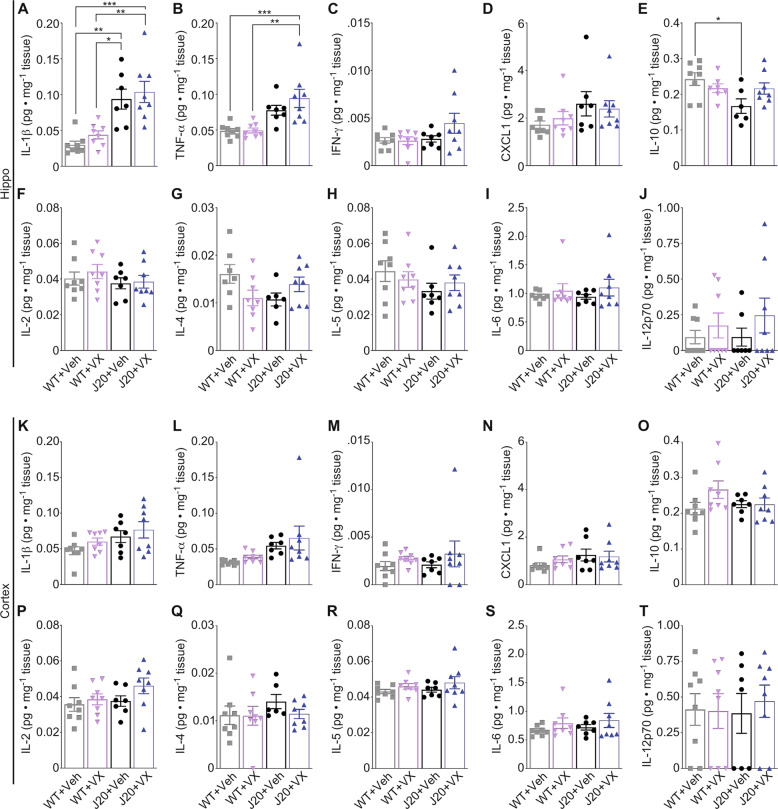
VX-765 treatment normalizes decreased IL-10 levels, but not increased Il-1β and TNF-α levels in 15-month-old J20 hippocampus. **A**–**J** Hippocampal protein levels for (**A**) IL-1β [*F*_genotype_ = 33.98, *p* < 0.0001], (**B**) TNF-α [*F*_genotype_ = 22.81, *p* < 0.0001], (**C**) IFN-γ, D CXCL1, (**E**) IL-10 [*F*_genotype_ = 5.21, *p* = 0.03; *F*_interaction_ = 4.95, *p* = 0.04], (**F**) IL-2, G IL-4, H IL-5, I IL-6, J IL-12p70. **K**–**T** Cortical protein levels for (**K**) IL-1β, (**L**) TNF-α, M IFN-γ, N CXCL1, (**O**) IL-10, P IL-2, (**Q**) IL-4, R IL-5, (**S**) IL-6, (**T**) IL-12p70. *N* = 8 WT + Veh, 8 WT + VX, 7 J20 + Veh, and 8 J20 + VX mice for hippocampus and cortex. One-way ANOVA, Bonferroni’s post-hoc for all panels. Bars represent mean ± SEM; symbols denote performances of individual mice. *p < 0.05, **p < 0.01, ***p < 0.001.

To further assess inflammatory markers, cytokine production in the hippocampus and cortex was quantitated with ELISA. Hippocampal IL-1β in 15-month-old vehicle-treated J20 increased compared to WT and, surprisingly, was unaltered with VX-765 (Fig. 4A). Hippocampal TNF-α was unaffected in vehicle-treated J20 but significantly increased after VX-765 (Fig. 4B). IFNγ (Fig. 4C) and CXCL1 (Fig. 4D) pro-inflammatory cytokines, previously shown to increase in 4-5 month-old J20 hippocampus [11], did not change across treatment groups in 15-month-old J20 hippocampus. Conversely, hippocampal anti-inflammatory IL-10 decreased in vehicle-treated J20 compared to WT and was normalized after VX-765 (Fig. 4E). No other differences were observed across treatment groups for hippocampal IL-2, IL-4, IL-5, IL-6, or IL-12p70 (Fig. 4F–J). In the cortex, no changes were detected for any of the cytokines (Fig. 4K–T). IL-18, which is also processed by Casp1, did not change in vehicle- or VX-765-treated J20 cortex (Fig. S4D). These results are consistent with the lack of effect of VX-765 on Iba1^+^-microglia levels in aged J20 hippocampus and cortex. However, the results suggest that IL-10 may contribute to VX-765’s beneficial effect against cognitive decline in J20 mice.

### VX-765 effects on hippocampal and cortex Aβ deposition and Aβ subspecies levels

**Fig. 5:**
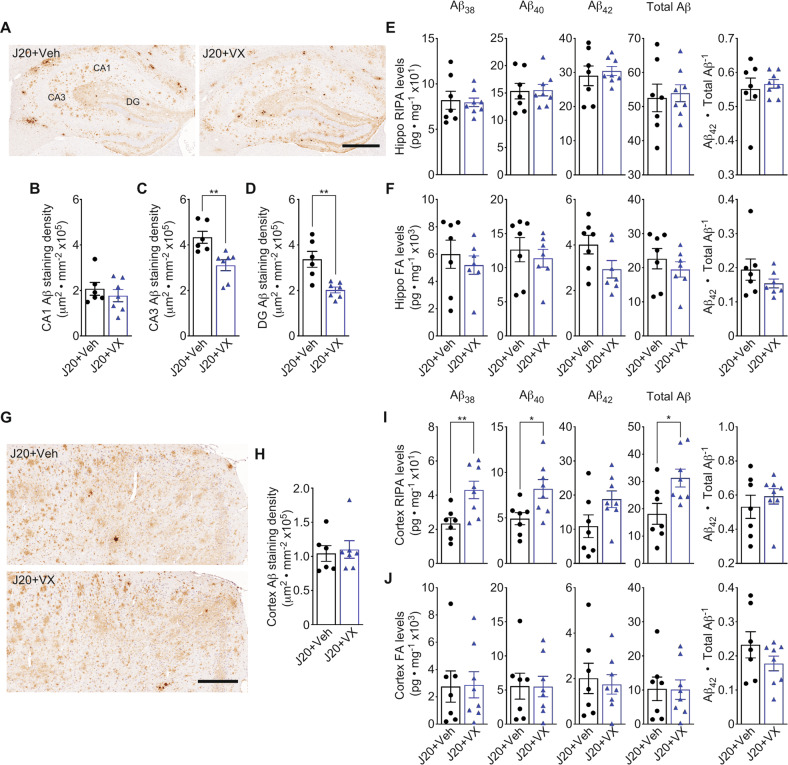
VX-765 reduces amyloid deposition, but not RIPA- or FA-soluble Aβ, in the hippocampus of 15-month-old J20 mice. **A** Representative Aβ^+^ immunostaining in the CA1, CA3, and DG of the hippocampus. Scale bar = 400 μm. **B**–**D** Aβ^+^ immunostaining density in the (**B**) CA1, (**C**) CA3 [*t* = 3.43, *p* = 0.005, unpaired t-test], and (**D**) DG [*t* = 3.88, *p* = 0.003, unpaired *t*-test] of the hippocampus. **E** RIPA-soluble and (**F**) formic-acid soluble Aβ_38_, Aβ_40_, Aβ_42_, total Aβ protein levels, and Aβ_42_/total Aβ ratio in the hippocampus. **G** Representative Aβ^+^ immunostaining (scale bar = 200 μm) and (**H**) density quantification in the cortex. **I** RIPA-soluble [*t*_Aβ38_ = 3.11, *p* = 0.008; *t*_Aβ40_ = 2.61, *p* = 0.02; *t*_total Aβ_ = 2.61, *p* = 0.02, unpaired *t*-test] and (**J**) formic-acid soluble Aβ_38_, Aβ_40_, Aβ_42_, total Aβ protein levels, and Aβ_42_/total Aβ ratio in the cortex. *N* = 6 J20 + Veh and 7 J20 + VX mice for immunostaining density quantifications (**B**–**D**, **H**), and *n* = 7 J20 + Veh and 8 J20 + VX mice for ELISA quantitation (**E**, **F**, **I**–**J**). Bars represent mean ± SEM; symbols denote performances of individual mice. *p < 0.05, **p < 0.01.

Transgenic human APP levels and processing into alpha- and beta-C-terminal fragments (CTF) were not altered by VX-765 in J20 (Fig. S5A–E). Hippocampal Aβ immunostaining density (Fig. 5A) did not change in the CA1 (Fig. 5B) but was decreased in the CA3 (Fig. 5C) and DG (Fig. 5D) of VX-765-treated J20. However, hippocampal Aβ levels, quantitated by MSD ELISA, were unaltered in VX-765-treated J20. Neither RIPA-soluble or formic acid (FA) soluble Aβ_38_, Aβ_40_, Aβ_42_, total Aβ, nor Aβ_42_/total Aβ ratio differed between vehicle- and VX-765 treated J20 mice (Fig. 5E, F).

Cortical Aβ immunopositivity did not decrease between vehicle- and VX-765-treated J20 (Fig. 5G, H). VX-765 increased RIPA-soluble Aβ_38_, Aβ_40_, and total Aβ, but did not change Aβ_42_ or Aβ_42_/total Aβ levels compared to vehicle-treated J20 (Fig. 5I). However, there were no changes in FA soluble Aβ_38_, Aβ_40_, Aβ_42_, total Aβ, or Aβ_42_/total Aβ between vehicle- and VX-765 treated J20 (Fig. 5J).

These results indicate that VX-765 only slightly alters hippocampal Aβ deposition and does not reduce biochemically-measured Aβ. Conversely, cortical Aβ deposition is not altered but RIPA-soluble total Aβ, Aβ_38_, and Aβ_40_ are increased.

## Discussion

To determine if VX-765 can correct cognitive deficits and AD-related pathologies in a more advanced disease stage, 12- to 15-month-old J20 mice were submitted to an identical VX-765 treatment regimen as that previously used on 5- to 8-month-old J20 [12]. J20 develop memory deficits at four months of age [29], therefore treatment in 12-month-old J20 began 8 months after memory impairment onset compared to just one month after onset in 5-month-old mice. VX-765 treatment of aged J20 significantly reversed cognitive impairment, decreased synaptophysin immunoreactivity, and decreased dendritic spine density without increased altering pro-inflammatory cytokine levels, GFAP^+^-astrocytes and Iba1^+^-microglia numbers. VX-765 slightly reduced hippocampal Aβ deposition without significantly reducing Aβ subspecies or total Aβ protein levels and did not alter cortical Aβ deposition but increased Aβ_38_, Aβ_40_, and total Aβ.

Aged mice did not respond as rapidly nor as completely to VX-765 than younger J20 treated at onset of cognitive deficits. VX-765 efficiently reversed NOR episodic memory deficits in 75% and 88% of 12- to 15-month-old J20 compared to vehicle-treated J20 after one (T1) and three (T2) weeks of VX-765, respectively. One-month WO reduced cognitive improvement to 38% of mice treated, but after re-introducing VX-765 (T3), 100% of J20 mice cognitively improved. This indicates that the cognitive response is due to VX-765 treatment, but deficits can return when VX-765 is removed. In comparison, in 5- to 8-month-old J20 [12], 100% of treated mice improved at T1, T2, and T3, despite a decline to 56% after WO. When compared to WT mice, NOR deficits in 12- to 15-month-old VX-765-treated J20 were normalized in 19% of mice at T1 and 87% of mice at T2. Cognitive deficits were normalized in only 25% of VX-765-treated J20 after WO and increased to 40% of mice after re-administration of VX-765. In the 5- to 8-month-old J20, cognitive deficits were normalized in 89% and 56% at T1 and T2, respectively, 11% after WO, and 100% of J20 mice at T3. These results show that aged mice respond more slowly to VX-765 than younger mice. Nevertheless, considering the more advanced stage of AD pathology and extent of time with cognitive deficits, the response is remarkable since all aged J20 mice cognitively improved with longer VX-765 treatments.

VX-765 improved spatial learning and memory more than episodic memory in aged J20 mice. Aged J20 showed a more pronounced improvement in learning during training and probe errors were normalized to WT with VX-765 after both T2 and WO. This differential response between episodic and spatial memory in aged J20 mice might be explained by the fact that episodic memory functions are almost exclusively hippocampal dependent, whereas spatial memory functions elicit cortical areas [32]. Higher Il-1β, TNF-α and total Aβ or Aβ subspecies in hippocampus compared to cortex indicate that AD-related pathologies may exacerbate loss of hippocampal function compared to cortical function.

Aβ does not appear to be crucial for cognitive deficits in these older J20 mice despite higher RIPA- and FA-soluble hippocampal Aβ accumulation than younger J20 [12]. A 25% reduction of Aβ deposits resembling plaques in hippocampal CA3 and DG without alteration of RIPA- or FA-soluble total Aβ or Aβ subspecies, suggests that Aβ unlikely accounts for the strong VX-765-mediated cognitive recovery of VX-765-treated J20. In contrast, VX-765 or *Casp1* KO in 5- to 8-month-old J20 significantly decreased hippocampal Aβ [11, 12]. However, consistent with the present study, Aβ levels remained unaltered in the hippocampus and cortex of VX-765-treated-pre-symptomatic J20 despite significantly delaying the onset of cognitive deficits [17]. Furthermore, Aβ has been demonstrated to be toxic to neuronal function via a multitude of pathways and mechanisms [33]. Therefore, our results suggest that it is not Aβ that causes cognitive deficits. These results are consistent with the inability to reverse or delay AD progression, duration, or severity with anti-Aβ vaccines despite their ability to remove brain amyloid [34]. Nevertheless, recently, human CSF Aβ_38_ levels have been associated with a lower progression of cognitive decline [35] and, here, RIPA-soluble cortical Aβ_38_ levels increased two-fold with VX-765. Therefore, we cannot exclude a positive effect of Aβ_38_ on older J20 cognition. The increase in cortical RIPA-soluble Aβ_40_ and total Aβ is unexpected in cognitively normalized J20 since these are normally associated with cognitive impairment [33].

The present results indicate that inflammation may not be the primary driver of cognitive impairment in aged J20 mice. Unexpectedly, Iba1^+^-microglia and GFAP^+^-astrocytes in older J20 remained elevated in cognitively normalized VX-765-treated aged J20. In contrast, an identical VX-765 treatment regimen and *Casp1* KO in 5- to 8-month-old J20 normalized Iba1^+^-microglia, elevated hippocampal TNF-α and CXCL1, and reversed or prevented the onset of cognitive impairment [11, 12]. Similarly, pre-symptomatic VX-765 treatment of J20 ablated microglial inflammation, and both hippocampal and cortical Iba1^+^-microglia correlated strongly with episodic memory decline [17]. Other studies observed the same positive association between microglial inflammation and cognitive deficits in APP/PS1 mice [13]. Fibrillar Aβ-associated immune mechanisms involving microglial NLRP3 and Casp1 have been proposed to drive AD risk, onset, and disease progression [36, 37]. Here, VX-765 reversed cognitive deficits without altering increased pro-inflammatory Iba1^+^-microglia, Il-1β, TNF-α, and GFAP^+^-astrocytes, thereby seemingly excluding inflammation as a driver of cognitive impairment. However, compared to the younger mice, not all VX-765-treated aged J20 returned completely to normal cognitive levels. It is therefore possible that microglial activation contributes to an exacerbation of cognitive deficits.

Alternatively, the anti-inflammatory cytokine, IL-10, may play a role in modulating cognition. Decreased J20 hippocampi IL-10 levels were normalized by VX-765. However, IL-10’s beneficial role in VX-765-treated J20 is inconsistent with previous results where genetically ablating *Il-10* in APP/PS1 mice increased synaptophysin levels, reduced Aβ accumulation, increased Iba1^+^-microglia and GFAP^+^-astrocytes, and improved cognition [38]. Furthermore, virally overexpressed Il-10 in the TgCRND8 AD mouse model exacerbated Aβ accumulation and worsened impaired memory [39].

Reversal of cognitive deficits with VX-765 is likely due to reversal of neuronal degeneration, which is induced via the Nlrp1 inflammasome-Casp1-Casp6 neurodegenerative pathway [1, 11, 40]. The fact that [1] the expression of the APP^SW/Ind^ is under a predominantly neuronal promoter in brain [2], the Nlrp1-Casp1-Casp6 pathway was validated by genetic ablation of either of these three genes in J20 mice [3], the activation of Nlrp1-Casp1-Casp6 in neurons leads to neurodegeneration, and [4] VX-765 normalize or improve cognition in J20 mice without a significant effect on Iba1^+^-microglia, Il-1β, and TNF-α, GFAP^+^-astrocytes, and Aβ levels support the hypothesis that neuronal degeneration drives cognitive deficits rather than Aβ and inflammation. Generally, it has been assumed that hippocampal neuronal death explains cognitive deficits in humans. While convincing neuronal death has been demonstrated in severe AD cases [41–54], few papers have reported neuronal loss in mild cognitive impairment (MCI) or mild AD [55]. Neuronal loss in MCI was observed in layer II of the entorhinal cortex [56], but not in the CA1 [49]. A recent extensive study of 14 brain regions in MCI and mild AD individuals, categorized by functional assessment staging scores of 3-4 and Braak stage III-V, reported 23–49% neuronal loss in the entorhinal cortex, CA1, subiculum, amygdala, thalamus, substantia nigra and dentate nucleus [52]. AD mouse models exhibiting cognitive decline without significant neuronal loss are often criticized as incomplete models of AD. In J20, CA1, but not CA3 neurons, decreased 10–30% at 3- to 9-months of age [57], but we did not observe loss of PCL neurons in 15-month-old J20 CA1. Furthermore, neuronal death as the cause of cognitive decline in these aged J20 can be excluded since loss of function is irreversible in cell death and VX-765 reversed both episodic and spatial memory impairment. It is more likely that a reversible type of degeneration, defined by loss of neuronal structure or function in the absence of cell death, and induced via the neuronal Nlrp1-Casp1-Casp6 pathway is responsible for cognitive deficits in J20. This would explain why VX-765 corrected cognitive impairment. Consistently, VX-765 mitigated J20 decreased dendritic spine density, decreased mature functional mushroom subtype, and increased immature stubby spines. In addition, synaptophysin levels in 15-month-old J20 hippocampal CA1, CA3, DG, and cortex decreased by 32%, 55%, 64%, and 37%, respectively, compared to WT. Reversal of cognitive deficits with VX-765 coincided with the return of normal hippocampal synaptophysin in the CA1 and improved synaptophysin levels in the CA3, DG, and cortex, respectively. The role of synaptophysin in cognition is supported by reduced NOR and spatial learning and memory performance in synaptophysin null mice [30] and a correlation between lower hippocampal synaptophysin levels and cognitive decline in AD [58]. Consequently, these results bring hope for an efficient treatment with VX-765 to improve cognition or prevent cognitive decline in more advanced AD stages.

In conclusion, these results indicate that inflammation and amyloid accumulation are not drivers of episodic or spatial memory deficits in J20 mice, although these pathologies may exacerbate cognitive deficits. Therefore, directly targeting neuronal degeneration pathways, rather than targeting AD-related pathologies, may provide more efficient treatments against age-dependent and AD-related memory impairment.

## Methods

### Animal studies

The Swedish (670/671_KM→NL_) and Indiana (717 _V→F_) human APP expressing J20 APP^Sw/Ind^ transgenic mouse (B6.Cg-*Zbtb20*^Tg(PDGF-APPSwInd/20lms)^/2Mmjax, stock No 006293, Jackson Laboratories, ME, USA) [59] was used for these experiments. J20 sperm and female C57BL/6 J wild-type (WT) mice were used with IVF to generate cohorts of J20 mice and their WT littermates. Mice were generated and aged to 11.5 months at the Institut de Recherche en Immunologie et en Cancérologie (IRIC) at the Université de Montréal. During this aging period, mice were housed 2–4 mice per cage in standard macrolon cages under a 12-h regular light/dark cycle, controlled environmental conditions, and ad libitum access to food and water. Approximately 2–3 weeks prior to experimentation (starting at 12-months of age), mice were transferred to the Lady Davis Institute (Jewish General Hospital, Montreal, QC) for habituation. Here, mice were also grouped housed (with their same cage mates during aging) under a 12-h reverse light/dark cycle with controlled environmental conditions and ad libitum food and water.

Three separate cohorts were used for these studies; two behavioral cohorts were tested approximately 4 months apart. A third cohort was used solely for Golgi-Cox analysis without behavioural testing. Both males and females were used for these studies. All animal protocols followed the Canadian Council on Animal Care guidelines approved by the Comité de Déontologie de l’Expérimentation sur les Animaux (CDEA, Université de Montréal) and McGill University Animal Care committee.

### Experimental design and VX-765 treatment

The Casp1 inhibitor, VX-765 (Adooq Bioscience, Irvine, CA, USA), was dissolved to 5 mg • ml^−1^ in 20% cremophor (Sigma-Aldrich, ON, Canada) in dH_2_0. Both WT and J20 mice received three injections per week administered IP of either 50 mg • kg^−1^VX-765 or 20% cremophor vehicle.

Behavioural experimentation and treatments started at 12-months of age. Mice were longitudinally assessed, first at baseline prior to treatment and then after 1) three injection treatments (T1), 2) an additional six injections (T2), 3) a four-week washout period with no treatment injections (WO), and 4) an additional three injection treatments after WO (T3) (Fig. 1A). Each mouse was randomly assigned to receive VX-765 or vehicle independent of baseline behavioural results and received a total of 12 injections by the end of the experimental paradigm. All animals were euthanized and randomly assigned to histological or biochemical analysis after the completion of T3 behaviour.

In a separate experiment, mice received only the first set of injections totaling 9 injections of either VX-765 or vehicle and euthanized at T2 for Golgi–Cox analysis. These mice did not undergo any behavioural testing.

During the 3-month behavioural testing period, 1VX-765-treated WT, 3 vehicle-treated J20, and 1 VX-765-treated J20 mice died but were kept in the behavioural analyses (Fig. S6). No mice were excluded from subsequent post-mortem analyses.

### Behavioural analyses

Each mouse underwent open field analysis followed by the novel object recognition (NOR) test 24 h later at each time point. The Barnes maze test was completed twice, the first was 24 h after the NOR test at T2 and WO. Investigators were blind to mouse genotype and treatment group during behavioural testing.

#### Open field

locomotor activity and hyperactivity were measured using an automated movement tracking system (HVS Image, Hampton, UK). Mice were placed in a plexiglass open field chamber measuring 40 × 40 cm with no ceiling and white floor and allowed to freely explore for 5 min. Total distance traveled, percentage time moving, and percentage time in the periphery (thigmotaxis) were measured for each mouse.

#### NOR

Mice were placed in the same open field chamber and pre-exposed to two identical objects, placed on the outer edges of the chamber, for 5 min. and returned to their cage. Two hours after pre-exposure, mice were placed back into the chamber and exposed to one familiar object and one novel object for an additional 5 min. During this test phase, the discrimination index [DI; (# of touches of novel object - # of touches of familiar object)/total touches] was assessed for each mouse. Novel object placement was counterbalanced for each treatment group. Since mice underwent NOR testing at multiple times, different objects were used so that mice were not exposed to an object more than once.

#### Barnes maze

The Barnes maze was comprised of a circular platform, 91 cm in diameter and elevated 90 cm from the floor, with 20 holes (each measuring 5 cm in diameter) evenly spaced on the outer edges. All holes were blocked except for one target hole which led to a recessed escape hatch underneath the Barnes platform. Spatial cues, bright light, and white noise were used to motivate mice to find the escape hatch. Adaptation phase: prior to testing, each mouse was allowed to explore the platform for 60 s to find the target escape hatch. Mice that did not find the escape hatch during this time were then guided to it and allowed to remain there for 90 s. Learning acquisition phase: Mice underwent training with four trials per day, approximately 15 min apart, for four consecutive days to find the target. Each trial lasted 180 s. Mice remained in the escape hatch for 60 s. Any mouse that did not find the target escape hatch after 180 s was guided there. Probe test: After learning to find the escape hatch, each mouse underwent a single 90 s trial to find the target, except this time the escape hatch was blocked. The latency and errors to reach the target, plus the number of “pokes” to each hole (target preference) was measured.

### Tissue processing

Mice earmarked for immunohistochemistry were anaesthetized with isoflurane and transcardially perfused with ice-cold saline followed with 4% paraformaldehyde in 0.1 M PBS (Sigma-Aldrich). Brains were dissected out, post-fixed in 10% neutral-buffered formalin (Thermo Fisher Scientific, ON, Canada) overnight at 4°C, and subsequently transferred into 70% EtOH for paraffin embedding and sectioning at 4 μm thickness.

For western blot and ELISA biochemical analyses, mice were anaesthetized with isoflurane and cervically dislocated. The hippocampus and cortex were immediately dissected out, frozen on dry ice, and stored at −80 °C until used. Brain proteins were extracted in 5x volume/weight with radioimmunoprecipitation assay (RIPA) buffer containing 50 mM Tris-HCl (pH 7.4), 1% NP-40, 150 mM NaCl, 0.25% Na-deoxycholate, 1 mM EDTA, and protease and phosphatase inhibitors (300 μM AEBSF, 2 μM leupeptin, 2.4 μM pepstatin A, 0.8 μM TLCK, 1 mM Na_3_VO_4_, 1 mM NaF, and 1 mM PMSF) on ice using a tissue homogenizer (OMNI International, Kennesaw, GA, USA). Samples were centrifuged at 20,000 g at 4 °C, the supernatant recovered, and protein was quantified with BCA. The RIPA-insoluble pellet went through further extraction in 3x volume/weight with 70% formic acid in dH_2_0 containing protease and phosphatase inhibitors. Samples were further centrifuged for 25 min, the supernatant recovered and the formic acid evaporated using a speed-vac, and resolubilized in 200 mM Tris-HCl, pH 7.5.

### Immunohistochemistry and quantification

Tissue immunostaining was performed using the Dako Autostainer Plus slide processor (Dako, ON, Canada) and EnVision Flex system (Agilent Technologies, ON, Canada). Slides underwent heat-induced antigen retrieval with either Tris-EDTA (pH 9.0) or Tris-sodium citrate (pH 6.5) buffer. Sections were blocked with Dual Endogenous Enzyme Block and Serum-Free Protein Block, and immunostained with the following antibodies diluted in antibody diluent (Agilent Technologies): 1:8000 mouse anti-synaptophysin (Sigma-Aldrich), 1:5000 mouse anti-NeuN (MAB377, Chemicon International, Temecula, CA, USA), 1:2000 rabbit anti-Iba1 (019-19741, Wako Chemicals, Richmond, VA, USA), 1:8000 rabbit anti-GFAP (Z-0334, Dako), and 1:2000 rabbit anti-Aβ_1-40_ [F25276, laboratory developed [12]]. Sections were incubated with an HRP-conjugated anti-mouse or anti-rabbit secondary antibody (Agilent Technologies), and immunoreactivity was revealed with diaminobenzidine and counterstained with hematoxylin (Dako). Sections were digitally scanned with MIRAX SCAN (Zeiss, ON, Canada) for analyses.

Iba1^+^-microglia cell densities were measured from the stratum oriens, stratum radiatum, and stratum lacunosum of the hippocampal CA1, and in the retrosplenial area (RS), primary and secondary motor areas, and primary somatosensory area of the cortex. The number of Iba1^+^ microglia per mm^3^ was quantitated using a modified version of an area-specific counting frame [60]. Changes in microglial morphology were qualitatively measured using pre-determined criteria (Fig. S4B) [12, 31]. Type I were considered ramified microglia with small cell bodies and long thin processes. Type II were identified as swollen ramified microglia with a slightly enlarged cell body and shorter processes. Type III were “reactive” microglia with a larger amoeboid-like morphology with either short or no processes. Type IV, or phagocytic microglia, were large, fragmented, structures often surrounding Aβ plaques.

NeuN^+^ neuronal cell density was measured in the hippocampal CA1 PCL between Bregma −1.22 to −1.82 mm. The number of NeuN^+^ cells per mm^3^ was quantitated using approximately 18–28 area-specific counting frames per mouse.

Immunopositive hippocampal synaptophysin and Aβ staining densities were measured separately in the stratum radiatum of the CA1, stratum lucidum of CA3 (CA3-SLu), and the molecular layer of the dentate gyrus (DG-M), and in the RS area, primary and secondary motor areas, and primary somatosensory areas of the cortex. GFAP staining density was measured only in the hippocampal CA1 and cortical RS, primary and secondary motor, and primary somatosensory areas.

All sampling was estimated across three to five sections using QuPath (University of Edinburgh, Scotland [61]) and ImageJ software (NIH, MD, USA). Investigators were blind to mouse genotype and treatment group during quantification and analysis. Representative images have only been cropped and did not undergo any post-processing.

### Golgi–Cox staining and analysis

Dendritic spine density and spine morphology were measured in a separate cohort of mice at T2. Brains were stained with the FD Rapid GolgiStain™ kit from FD NeuroTechnologies (Columbia, MD, USA), according to the manufacturer’s instructions. Mice were isoflurane anaesthetized, euthanized with cervical dislocation, and the brains dissected out and rinsed with dH_2_O. Brain tissues were incubated in impregnation solution for 2 weeks followed by solution C for 3 days in the dark at room temperature. Brains were sectioned at 100 μm, mounted on gelatin-coated slides (FD NeuroTechnologies), and stained according to the manufacturer’s protocol.

Sections were digitally scanned with MIRAX SCAN (Zeiss) using a 40x objective focusing particularly on dorsal hippocampal CA1 pyramidal dendrites in the SR. Approximately 10–15 neurons were looked at across 3–5 sections. Within each neuron, 2–4 dendrites with lengths ranging 15–50 μm were analyzed. Dendritic spines were manually counted and categorized into three groups based on morphology: mushroom spines with a large head and a narrow neck, stubby spines with a large head but lacking a neck, and thin spines with a small head and a narrow neck. Investigators were blind to mouse genotype and treatment group during analysis.

### Western blot and quantification

Protein samples (20 μg) were separated on 4–12% NuPAGE Bis-Tris gels (Invitrogen) or 4–20% TGX gels (Bio-Rad, Mississauga, ON, Canada) by polyacrylamide gel electrophoresis (PAGE) and transferred onto PVDF membranes (Bio-Rad). Membranes were blocked in 5% non-fat dry milk in Tris-buffered saline with 0.1% Tween-20. Membranes with proteins run on NuPage gels were probed with the following primary antibodies in PBS blocking buffer (ThermoFisher): 1:500 mouse anti-human Aβ_1-16_ (6E10) (803001,Biolegend, San Diego, CA, USA) and 1:2000 rabbit anti-APP C-terminus (A8717, Sigma-Aldrich, Oakville, ON, Canada). Membranes with proteins run on TGX gels were probed with the following: 1:5000 mouse anti-PSD95 (75-028, Neuromab, California, USA) and 1:2000 mouse anti-SNAP25 (SMI-81R-100, Biolegend). All membranes were incubated with 1:10 000 mouse anti-β-actin (A5441, Sigma-Aldrich). Immunoreactivity was detected using 1:5000 HRP-linked anti-mouse (Jackson ImmunoResearch Laboratories, West Grove, PA, USA) or anti-rabbit (Agilent Technologies) secondary antibodies, followed with incubation with enhanced chemiluminescence (ECL) (GE Healthcare Life Sciences, Mississauga, ON, Canada). Immunoreactive bands were visualized with the ImageQuant LAS 4000 imaging system (Fujifilm USA Valhalla, NY, USA). Densitometry of bands were analyzed with Image J (NIH). All protein levels were normalized to β-actin and expressed as a % of the average obtained for WT or J20 mice. Uncropped, original western blots of representative images presented in this manuscript are provided in the supplemental material file.

### ELISA

ELISA samples were prepared from RIPA and formic acid samples described above. Pro- and anti-inflammatory cytokines, and Aβ levels in mouse brains were quantified using ELISA kits from Meso Scale Discovery (Rockville, MD, USA). Inflammatory cytokines were quantified with a ten-plex mouse pro-inflammatory panel I which measures measured IL-1β, TNF-α, CXCLI, IFN-γ, IL-2, IL-4, IL-5, IL6, IL-10, and IL12p70. RIPA- and formic-acid soluble Aβ was measured using the multiplex Aβ 6E10 panel, which analyzed Aβ_38_, Aβ_40_, and Aβ_42_. Approximately 100–140 μm and 40–70 μm of protein was used to measure pro-inflammatory cytokines and Aβ, respectively. Samples and standards were run in duplicate and prepared according to the manufacturer’s protocols.

### Statistical analyses

In the discussion, behavioural comparisons determining if individual mice improved were assessed by whether an individual mouse behavioural scores were outside the mean ± one standard deviation of the J20 + Veh treatment group. Similarly, mouse behaviour was deemed normalized when individual mouse scores were within the mean ± one standard deviation of WT + Veh.

Sample sizes used here were based on previous behavioural studies that showed the minimum number of mice needed to show a significant deficit in J20 mice. Sample sizes and statistical analyses used (t-test, two-way ANOVA, and post-hoc comparisons) are all indicated in the figure or figure legends. All values are expressed as the mean ± SEM, with F and *p* values indicated in the figure legends. All statistical analyses were conducted using GraphPad Prism 8.0 software (GraphPad Software, San Diego, CA, USA).

## Supplementary information

Supplemental Figures

Supplemental Material: Original Western Blots

Reproducibility Checklist

## Data Availability

All data presented in this manuscript and supplementary file are readily available. Uncropped, original western blots of representative images are provided in a supplementary file. Microscope slides and digital scans of immunohistological staining are available upon request. QuPath Software (v. 0.3.0.) to view digital scans can be found on the following website: https://qupath.github.io. Any additional information is available upon request. Source data are provided with this manuscript.
