# Supplementary material for: Caspase-1 inhibition improves cognition without significantly altering amyloid and inflammation in aged Alzheimer disease mice
Source: Cell Death Dis. 2022 Oct 11;13(10):864. doi: 10.1038/s41419-022-05290-x (PMC9553979; doi:10.1038/s41419-022-05290-x)
Supplement: Supplementary file 1 — Supplemental Figures [file 41419_2022_5290_MOESM1_ESM.pdf]

Supplemental Material for:

Caspase-1 inhibition improves cognition without significantly altering amyloid and inflammation in aged Alzheimer disease mice

Joseph Flores[1], Marie-Lyne Fillion[1], and Andréa LeBlanc[1,2]

[1]Lady Davis Institute for Medical Research at Jewish General Hospital, Montréal, Québec, Canada

[2]Department of Neurology and Neurosurgery, McGill University, Montréal, Québec, Canada

*Corresponding author: Andrea LeBlanc, PhD, Bloomfield Center for Research in Aging, Lady Davis Institute for Medical Research, Sir Mortimer B Davis Jewish General Hospital, 3755 ch. Côte Ste-Catherine, Montréal, QC, Canada H3T 1E2. Tel.: +1 (514) 340 8222 ext 25303. e-mail address: andrea.leblanc@mcgill.ca

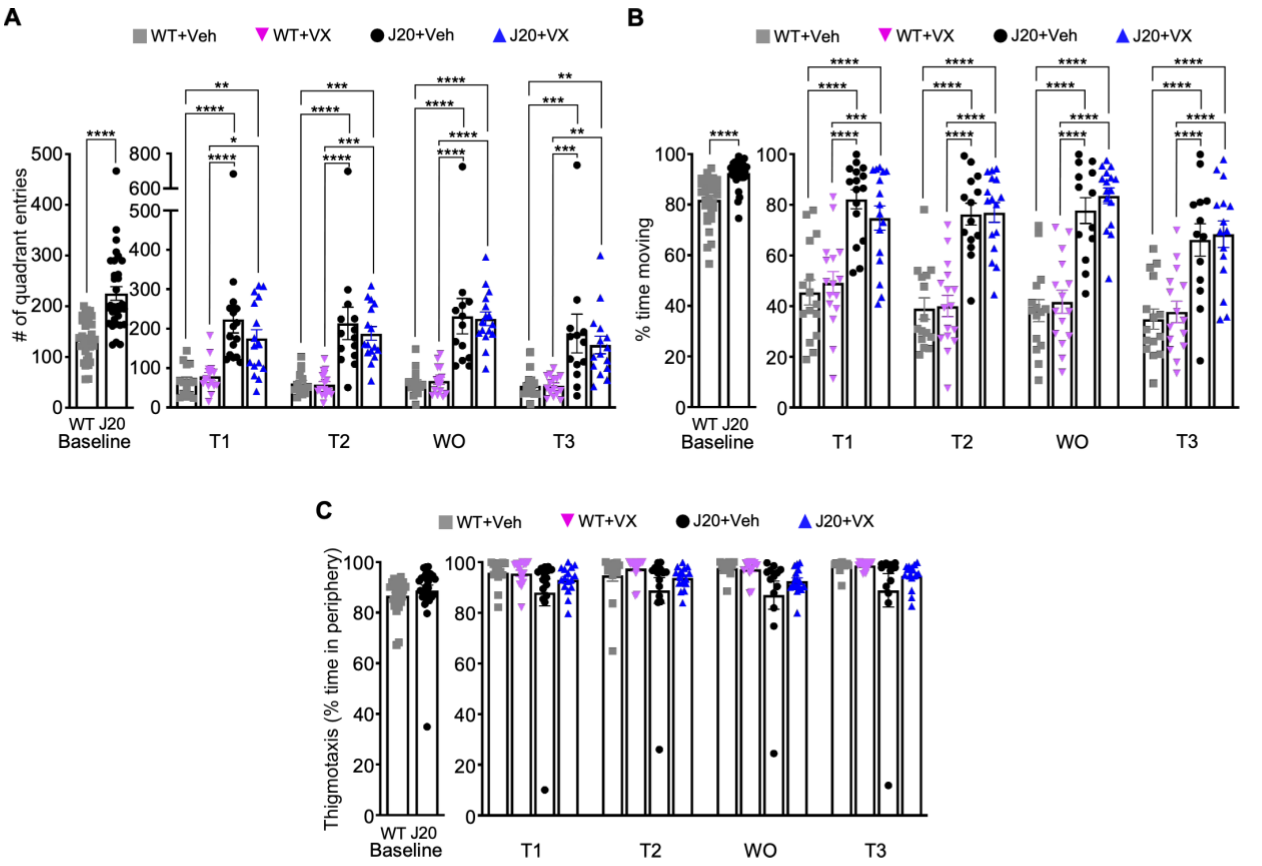

Fig. S1. **VX-765 does not reverse open field behaviour in aged J20 mice. A** Number of quadrant entries in the open field task at baseline [$t = 5.91$, $p < 0.0001$, unpaired t-test] and at post-treatment T1 (3 injections), T2 (9 total injections), WO (1 month washout), and T3 (12 total injections) [$F_{\text{time-point}} = 7.38$, $p = 0.0001$; $F_{\text{treatment}} = 14.98$, $p < 0.0001$; $F_{\text{interaction}} = 2.21$, $p = 0.02$, two-way ANOVA, Bonferroni's post-hoc]. **B** Percentage time moving in the open field task at baseline [$t = 5.62$, $p < 0.0001$, unpaired t-test] and at post-treatment T1, T2, WO, and T3 [$F_{\text{time-point}} = 11.26$, $p < 0.0001$; $F_{\text{treatment}} = 30.13$, $p < 0.0001$, two-way ANOVA, Bonferroni's post-hoc]. **C** Percentage time in the periphery, or thigmotaxis, in the open field task at baseline and at post-treatment T1, T2, WO, and T3. Bars represent mean ± SEM; symbols denote performances of individual mice. * $p < 0.05$, ** $p < 0.01$, *** $p < 0.001$, **** $p < 0.0001$.

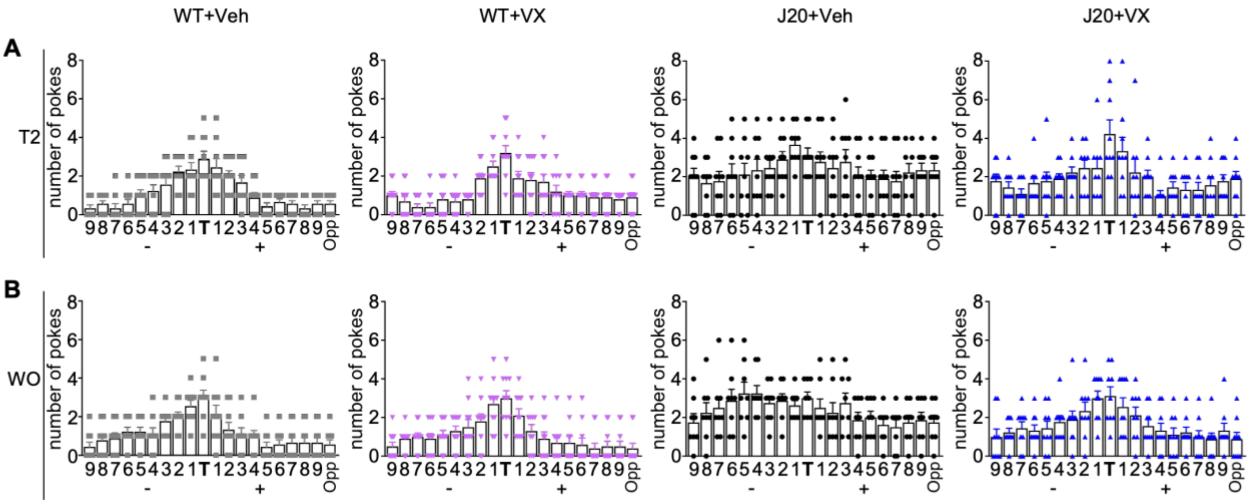

Fig. S2. VX-765 reverses Barnes maze deficits in aged J20 mice. A-B Barnes maze probe at (**A**) T2 and (**B**) WO showing distribution of nose "pokes" for each hole where (T) indicates target hole, +1 to 9 and – 1 to 9 indicates successive holes to the right and left of the target hole, respectively, and (Opp) indicates the hole directly opposite the target hole. Bars represent mean ± SEM; symbols denote performances of individual mice.

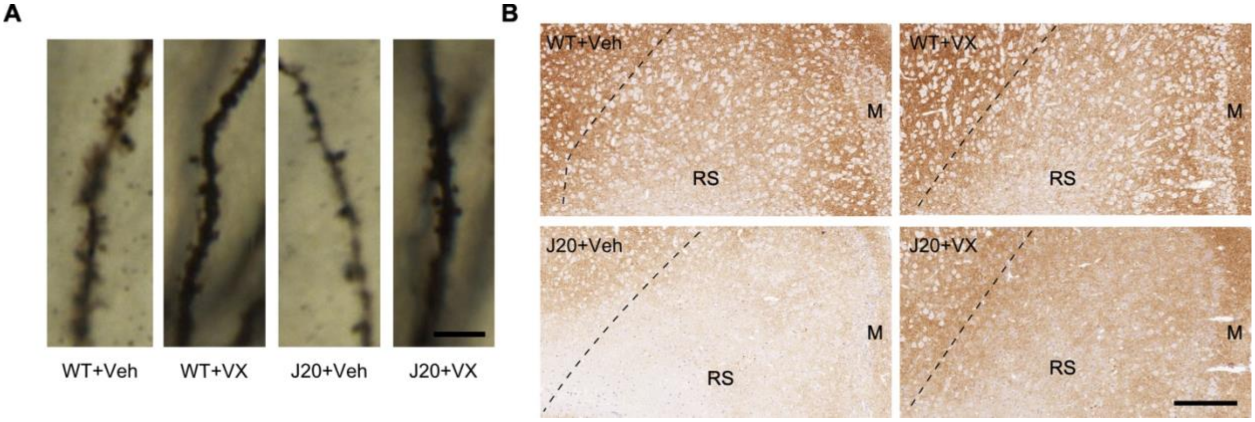

Fig. S3. VX-765 reverses loss of synaptophysin levels in the cortex of 15-month-old J20 mice. A Representative Golgi-Cox-stained dendritic spines in the hippocampal CA1 SR. Scale bar = 5 μm. **B** Representative micrographs of synaptophysin staining from the midline (M) to the retrosplenial (RS) area of the cortex. Scale bar = 200 μm.

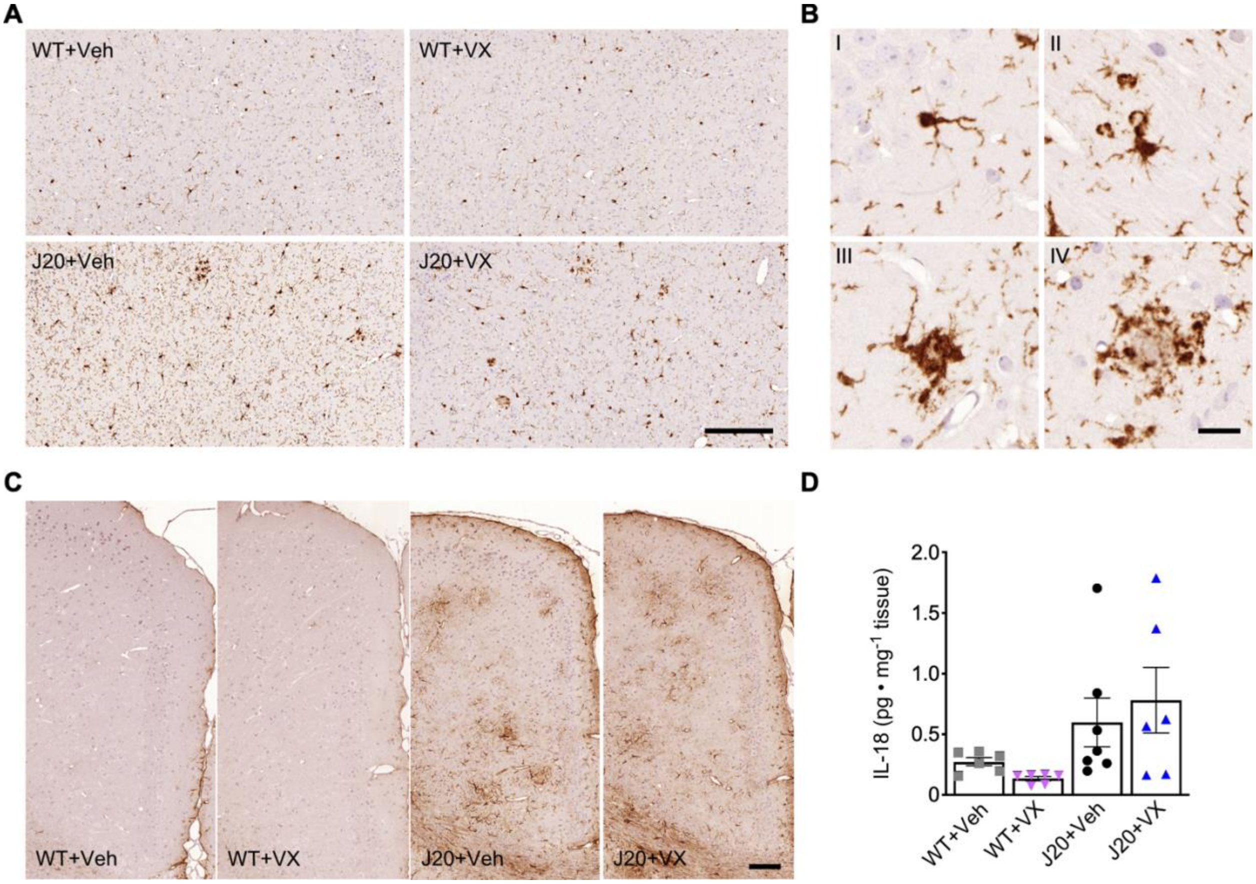

Fig. S4. VX-765 does not reverse inflammation in the cortex of 15-month-old J20 mice. A Representative micrographs of Iba1$^+$ microglial staining in the retrosplenial and motor areas of the cortex. Scale bar = 200 μm. **B** Iba1$^+$ micrographs representing type I, II, III, and IV microglial subtypes. Scale bar = 20 μm. **C** Representative GFAP$^+$ astrocyte staining in the retrosplenial cortex. Scale bar = 100 μm. **D** IL-18 cortical protein levels measured with sandwich ELISA. Bars represent mean ± SEM; symbols denote performances of individual mice.

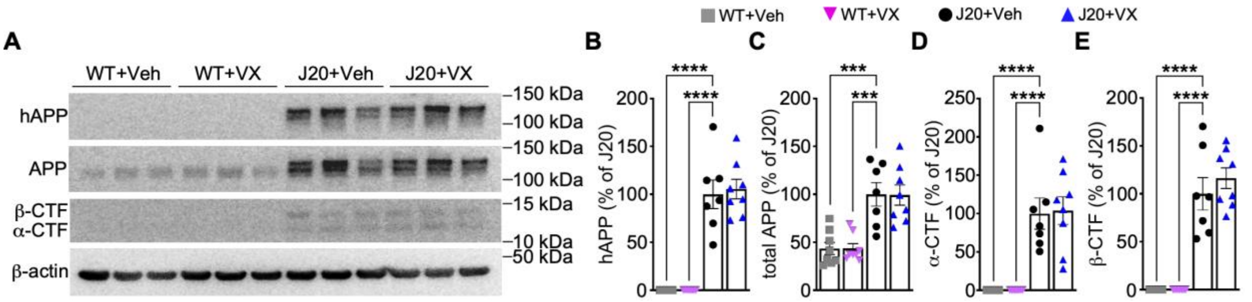

Fig. S5. VX-765 does not affect APP and CTF in the cortex of 15-month-old J20 mice. A Representative western blot images and **B-E** quantification of **B** human APP (hAPP) [$F_{genotype} = 146.3$, $p < 0.0001$], **C** total mouse + human APP [$F_{genotype} = 40.65$, $p < 0.0001$], **D** alpha (α) [$F_{genotype} = 61.32$, $p < 0.0001$] and **E** beta (β) [$F_{genotype} = 131.9$, $p < 0.0001$] C-terminal fragments (CTF) in the cortex. Two-way ANOVA, Dunnett's post-hoc compared to J20+Veh for (B-E). Bars represent mean ± SEM; symbols denote performances of individual mice. *** $p < 0.001$, **** $p < 0.0001$.

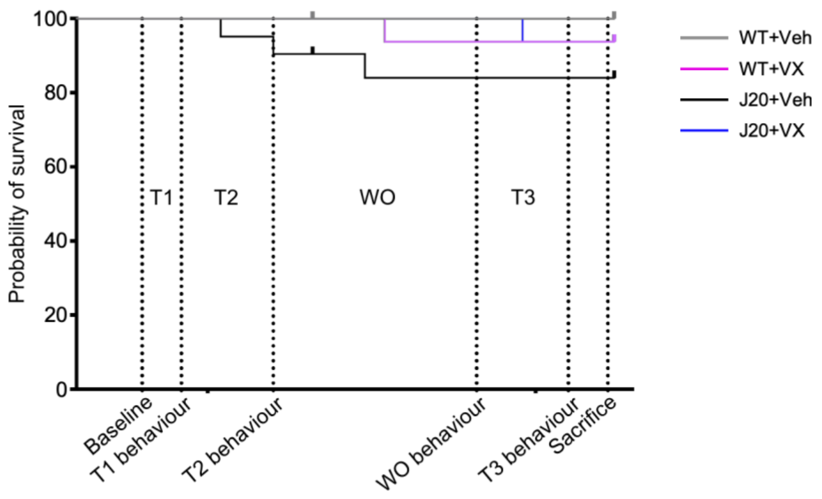

Fig. S6. VX-765 does not affect survival in 15-month-old J20 mice. Survival curve of WT and J20 mice during experimental paradigm. No significant differences found when comparing survival curves of the different treatment groups.
