# Supplementary material for: Caspase-1 inhibition improves cognition without significantly altering amyloid and inflammation in aged Alzheimer disease mice
Source: Cell Death Dis. 2022 Oct 11;13(10):864. doi: 10.1038/s41419-022-05290-x (PMC9553979; doi:10.1038/s41419-022-05290-x)
Supplement: Supplementary file 2 — Supplemental Material: Original Western Blots [file 41419_2022_5290_MOESM2_ESM.pdf]

Supplemental Material

Original western blots for:

Caspase-1 inhibition normalizes cognition without significantly altering amyloid and inflammation in aged Alzheimer disease mice

Joseph Flores, Marie-Lyne Fillion, and Andréa LeBlanc

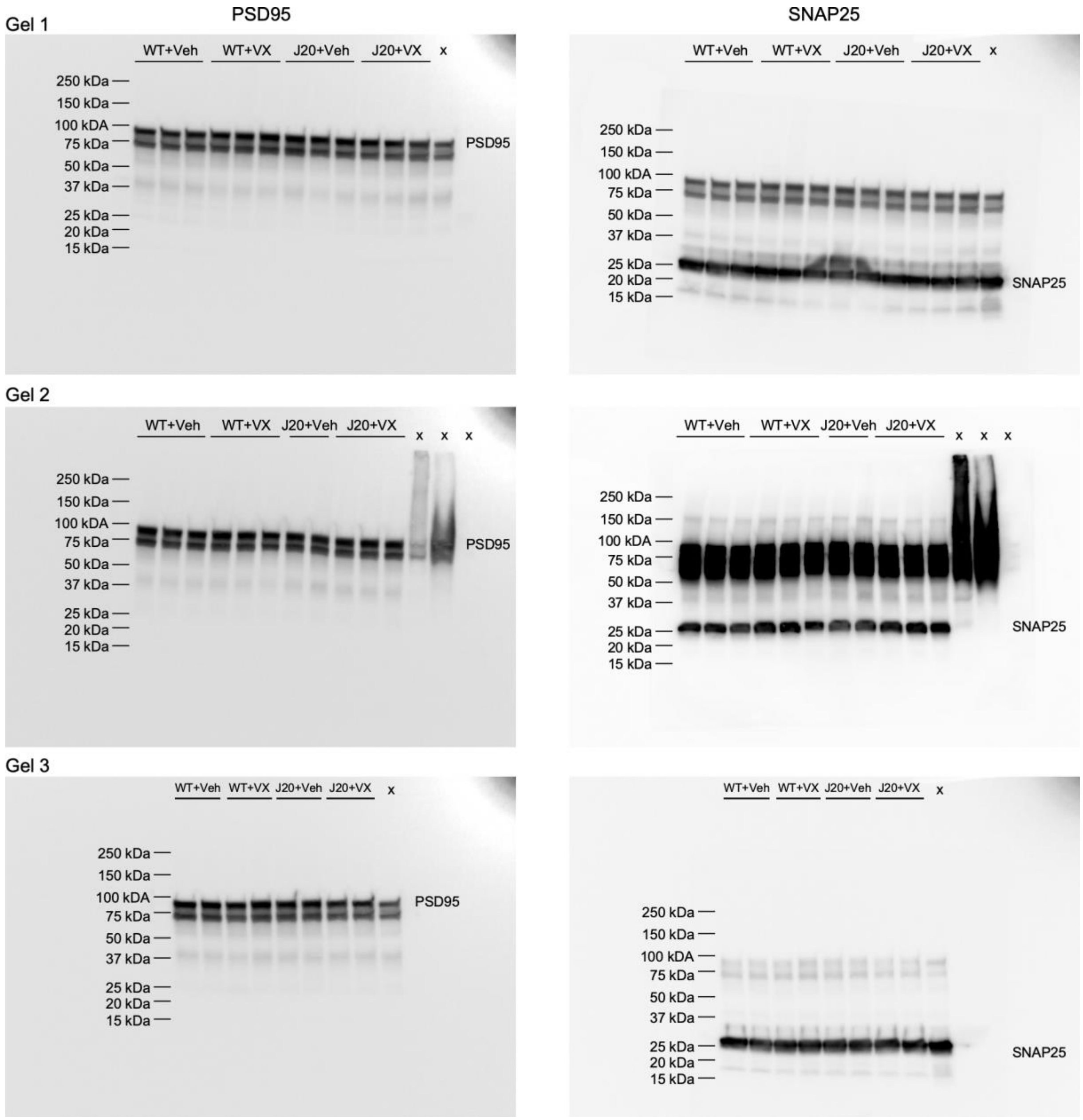

Original PSD95 and SNAP25 western blots for Fig. 2L,M quantification. Gel 3 used as representative image in Fig. 2K. Lanes marked ‘x’ are control lanes for other antibodies probed on these membranes but are not presented here.

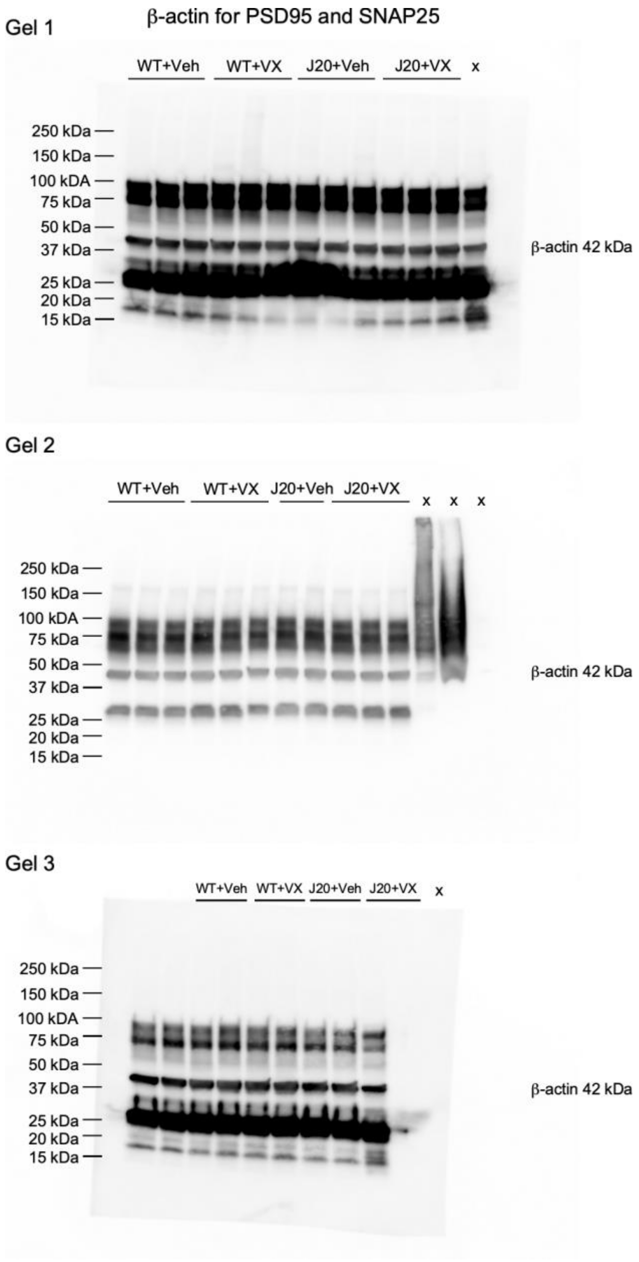

Original β-actin western blot for Fig. 2L,M quantification. β-actin Western blots for PSD95 and SNAP25 quantification. Gel 3 used as representative image in Fig. 2K. Lanes marked ‘x’ are control lanes for other antibodies probed on these membranes but are not presented here.

APP Representative Image

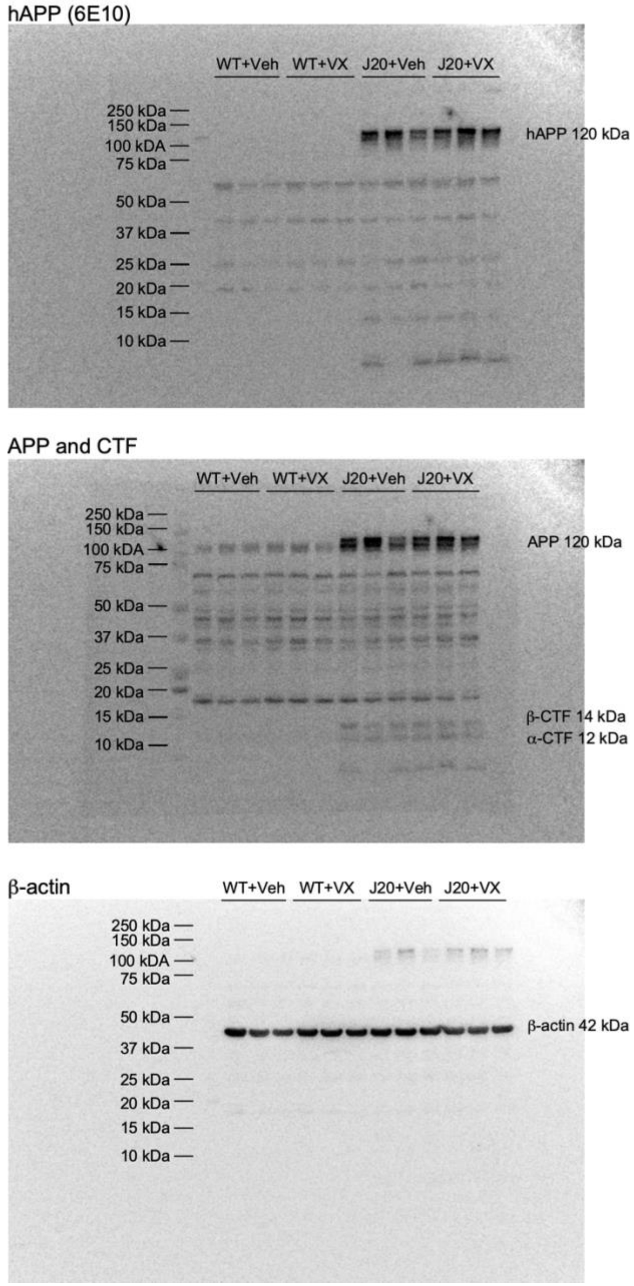

Original APP western blot of representative image seen in Fig. S5A.

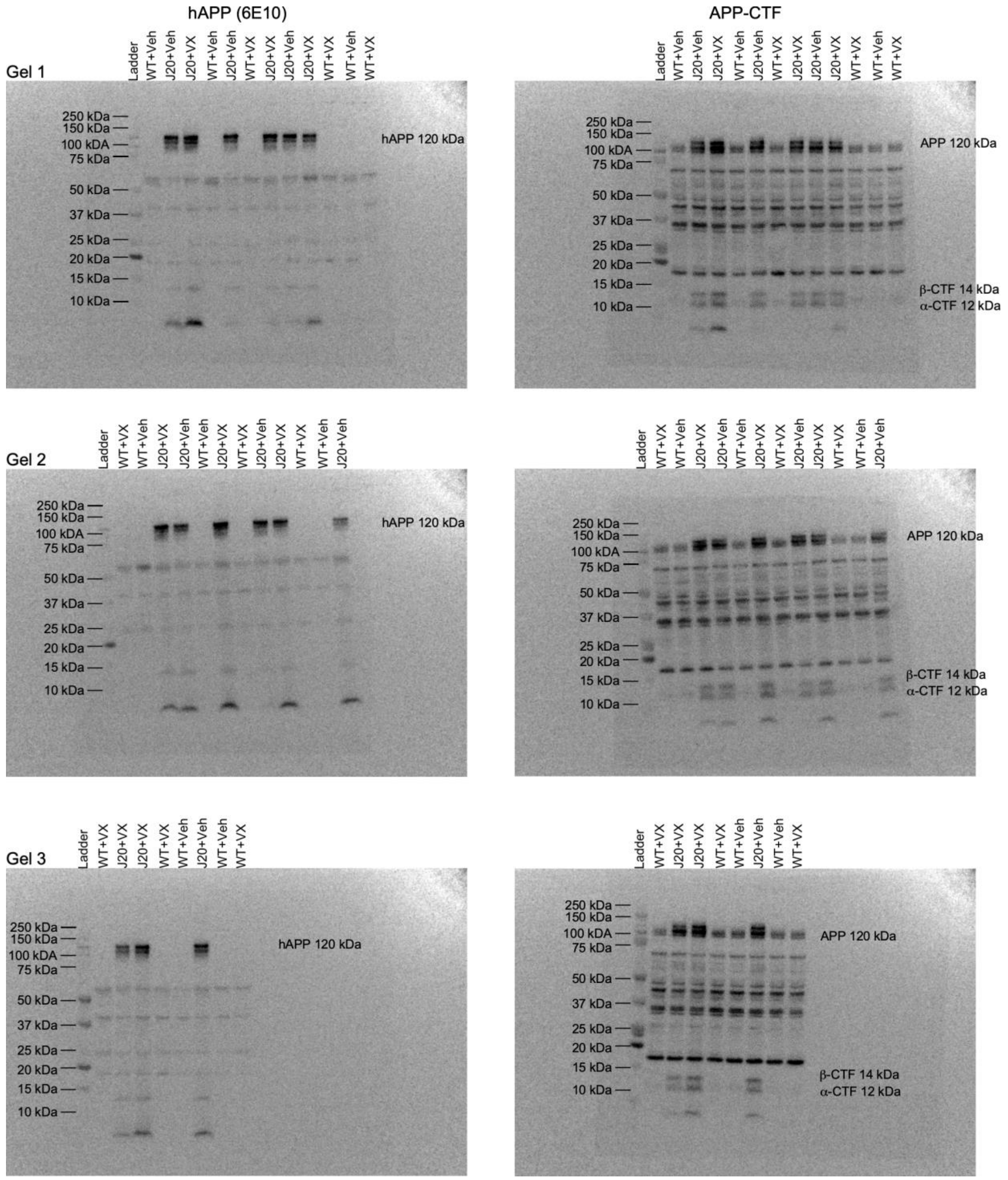

Original hAPP (6E10) and APP, α-CTF, β-CTF western blots for Fig. S5B-E quantification. Samples from all three blots were re-run on different gel to form representative image (Fig. S5A).

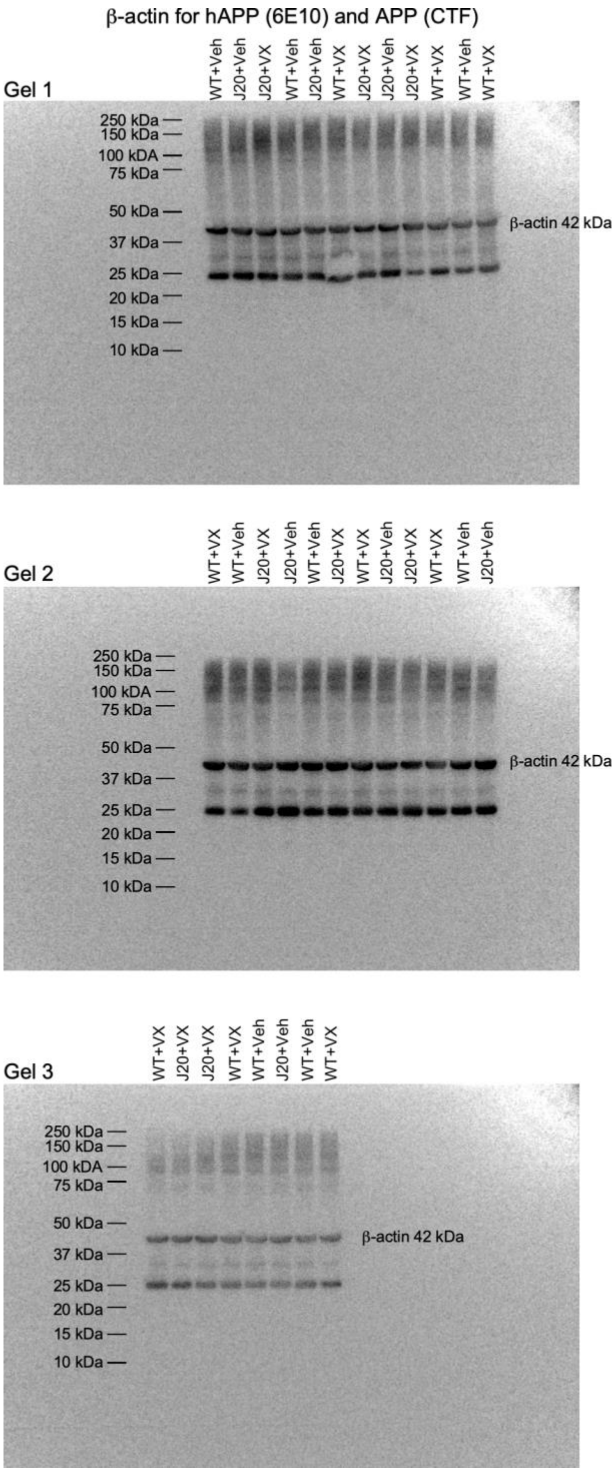

Original β-actin western blots for Fig. S5B-E quantification. Samples from all three blots were re-run on different gel to form representative image (Fig. S5A).
